# Cutavirus in Cutaneous Malignant Melanoma

**DOI:** 10.3201/eid2302.161564

**Published:** 2017-02

**Authors:** Sarah Mollerup, Helena Fridholm, Lasse Vinner, Kristín Rós Kjartansdóttir, Jens Friis-Nielsen, Maria Asplund, Jose A.R. Herrera, Torben Steiniche, Tobias Mourier, Søren Brunak, Eske Willerslev, Jose M.G. Izarzugaza, Anders J. Hansen, Lars P. Nielsen

**Affiliations:** University of Copenhagen, Copenhagen, Denmark (S. Mollerup, H. Fridholm, L. Vinner, K.R. Kjartansdóttir, M. Asplund, T. Mourier, S. Brunak, E. Willerslev, A.J. Hansen);; Technical University of Denmark, Kongens Lyngby, Denmark (J. Friis-Nielsen, J.A.R. Herrera, S. Brunak, J.M.G. Izarzugaza);; Aarhus University, Aarhus, Denmark (T. Steiniche);; Statens Serum Institut, Copenhagen (L.P. Nielsen);; Aalborg University, Aalborg, Denmark (L.P. Nielsen)

**Keywords:** Cutavirus, bufavirus, viruses, cancer, malignant melanoma, protoparvovirus, human, sequencing, metagenomics

## Abstract

A novel human protoparvovirus related to human bufavirus and preliminarily named cutavirus has been discovered. We detected cutavirus in a sample of cutaneous malignant melanoma by using viral enrichment and high-throughput sequencing. The role of cutaviruses in cutaneous cancers remains to be investigated.

Parvoviruses are small nonenveloped DNA viruses with a single-stranded linear genome of ≈5 kb. In 2016, a novel species within the *Protoparvovirus* genus was discovered in fecal samples from children with diarrhea in Brazil and subsequently detected in samples of mycosis fungoides lesions (cutaneous T-cell lymphoma) of patients in France (*1*). This virus, provisionally named cutavirus, shows highest identity to the human bufaviruses of the *Primate protoparvovirus 1* species. Bufaviruses are found in human fecal samples in low percentages (*2*–*7*). Using viral enrichment methods, we detected a cutavirus strain in an additional type of cancer, cutaneous malignant melanoma, further expanding the range of tissue types harboring cutaviruses and adding to the knowledge of the human virome.

We subjected a clinical sample of a cutaneous malignant melanoma lesion from a patient in Denmark to enrichment of virion-associated nucleic acids and enrichment of circular DNA molecules, followed by high-throughput sequencing (Technical Appendix). BLASTn (https://blast.ncbi.nlm.nih.gov/Blast.cgi?CMD=Web&PAGE_TYPE=BlastDocs&DOC_TYPE=Download) analysis originally identified contigs related to human bufaviruses in de novo assembled contigs from both datasets. In light of the recently published cutavirus genomes (*1*), we compared these sequences with the cutaviruses and found high similarity to the cutaviruses. From overlapping contigs, we obtained the 4,452 bp (from start nonstructural protein 1 [NS1] to end viral protein 1 [VP1]) near-complete genome of a novel cutavirus strain, CutaV CGG5–268 (GenBank accession no. KX685945). Similar to the other cutavirus genomes, CutaV CGG5–268 included NS1 and VP1 open reading frames (ORFs) encoding proteins of 659 aa and 707 aa, respectively. The CutaV CGG5–268 sequence also contained the small putative 333-nt middle ORF, starting at position 2021, and a 270-nt ORF located within the VP2 coding region, starting at position 2768. Further testing is required to determine whether these ORFs encode proteins.

We performed phylogenetic analysis based on the NS1 and VP1 amino acid sequences (Figure). Because 4 of the 7 published cutavirus genomes contain partial NS1 sequences, we included only 3 cutavirus strains in the phylogenetic analysis of NS1 . NS1-based analysis placed CutaV CGG5–268 closest to CutaV FR-F identified in a mycosis fungoides patient in France, whereas VP1-based analysis placed CutaV CGG5–268 closest to CutaV BR-450 identified in the feces of a child in Brazil.

**Figure:**
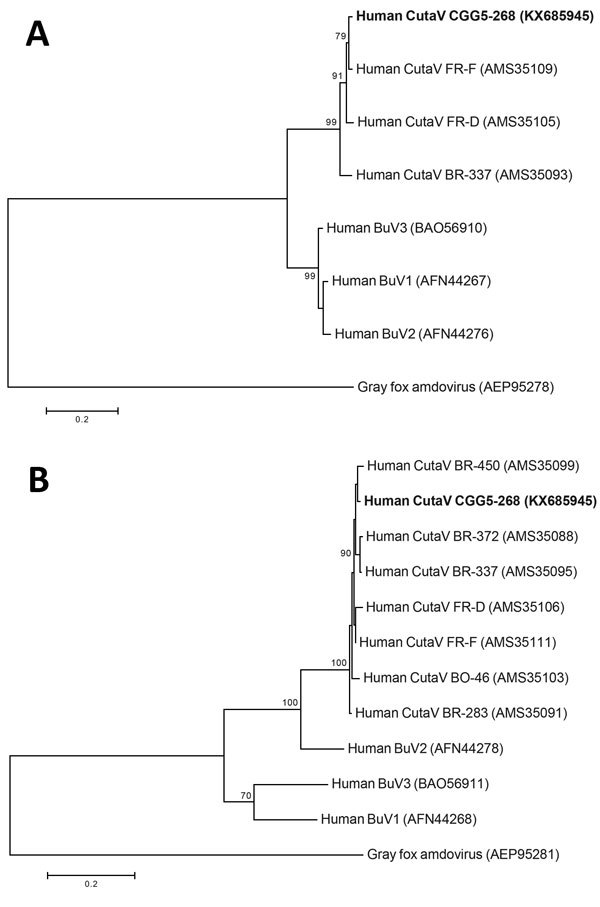
Phylogenetic analysis of human cutaviruses (CutaV) and bufaviruses (BuV) based on the full nonstructural protein 1 (A) and viral protein 1 (B) amino acid sequences. The trees were constructed by the maximum-likelihood method with 100 bootstrap replicates. Gray fox amdovirus was used as an outgroup. Bold indicates novel CutaV strain (CGG5–268) from this study. Scale bars indicate amino acid substitutions per position.

Cutaviruses were discovered in human fecal samples by use of metagenomics and subsequently detected in 4 of 17 samples of mycosis fungoides lesions; however, 21 skin samples, including samples from skin cancers and parapsoriasis lesions, tested negative for cutavirus (*1*). Our discovery of cutavirus in a sample of cutaneous malignant melanoma shows that extraenteric presence of cutaviruses is not limited to skin infiltrated by neoplastic T cells. The detection of cutaviral DNA after virion enrichment may indicate viral replication taking place in the affected tissue. Human bufaviruses have so far been detected only in fecal samples, predominantly from patients having diarrhea or gastroenteritis, and in only 0.27%–4% of samples (*2*–*8*). Another virus of the *Parvoviridae* family, human parvovirus B19, is shown to persist in multiple tissue types, in most cases without an established correlation to disease (*9*). Animal protoparvoviruses have also been detected in several sample types, as discussed elsewhere (*1*). Thus, future studies may reveal an expanded range of tissue types harboring cutaviruses. So far, cutaviruses have only been detected in the tissues investigated, and their direct involvement in disease has not been established. One limitation of this study is the lack of healthy controls for assessing whether cutavirus can also be detected in healthy skin. Furthermore, screening of a larger number of samples is necessary to determine the prevalence of cutavirus in malignant melanoma.

In 9 additional melanoma samples investigated in our laboratory, we did not identify contigs with similarity to those of cutavirus or bufavirus. All 10 samples were tested for cutaviral DNA by real-time PCR, but only the sample in which the cutaviral contigs were detected had positive results (Technical Appendix). We can only speculate regarding the cell tropism of cutaviruses; nevertheless, our study opens the possibility that cutaviruses replicate in melanocytes, which are present in the epidermal layers of the skin, where cutavirus DNA was detected by in situ hybridization (*1*). Melanocytes are also present in low numbers in the enteric epithelium, where melanomas can occur, though rarely (*10*). However, the cell tropism and potential pathogenicity of human protoparvoviruses remain to be investigated.

Technical Appendix Additional methods and details of results of testing showing cutavirus in a human cutaneous malignant melanoma biopsy sample.
