# Supplementary material for: Cutavirus in Cutaneous Malignant Melanoma
Source: Emerg Infect Dis. 2017 Feb;23(2):363–5. doi: 10.3201/eid2302.161564 (PMC5324802; doi:10.3201/eid2302.161564)
Supplement: Technical Appendix — Additional methods and details of results of testing showing cutavirus in a human cutaneous malignant melanoma biopsy sample. [file 16-1564-Techapp-s1.pdf]

Article: http://dx.doi.org/10.3201/eid2302.161564

Recently Discovered Cutavirus in Cutaneous Malignant Melanoma

Technical Appendix

Methods

Patient Sample and Ethics Statement

The human cutaneous malignant melanoma biopsy was obtained from the Department of Pathology, Aarhus University Hospital, Aarhus, Denmark. Sample collection, handling, and analysis were performed under the ethical protocols H-2–2012-FSP2 (Regional Committee on Health Research Ethics) and case no. 1304226 (National Committee on Health Research Ethics). In accordance with national legislation (Sundhedsloven), the sample was processed anonymously.

Enrichment of Small Circular DNA Molecules

Enrichment of circular DNA was performed as described (*1*). Briefly, total DNA was extracted from the sample using the QIAamp DNA Mini kit (QIAGEN, Hilden, Germany) following the manufacturer's instructions. Linear dsDNA was digested using 30 U Plasmid-Safe ATP-Dependent DNase (Epicentre, Illumina, San Diego, CA, USA) in the presence of 4 mM ATP for 3 h at 37°C. The remaining DNA was amplified for 16 h using the REPLI-g Midi Kit (QIAGEN) according to the manufacturer's instructions. Two µg of DNA was fragmented by the Bioruptor NGS (Diagenode, Liege, Belgium) to an average length of 300 bp. The sequencing library was prepared with NEBNext reagents (E6070) (New England BioLabs, Ipswich, MA, USA) with some modifications.

Enrichment of Virions

Enrichment of encapsidated nucleic acids was performed as described (*2*,*3*). Briefly, the biopsied tissue was homogenized in cold PBS using the TissueLyser II (Qiagen). The homogenate was centrifuged for 2 min at 800 × *g* to remove tissue debris and the supernatant

was then filtered through a 5-μm centrifuge filter (Millipore, Darmstadt, Germany). The filtrate was nuclease digested to remove unprotected nucleic acids with 14 μL TURBO DNase (2U/μL) (Ambion, Thermo Fisher Scientific, Foster City, CA, USA), 12 μL Baseline-ZERO DNase (1U/μL) (Epicentre), 16 μl RNase Cocktail Enzyme Mix (Ambion), and 40 μL 10 × TURBO DNase buffer in a total volume of 400 μL, and incubated at 37°C for 2 h. Nucleic acids from the enriched sample were extracted with the High Pure Viral RNA Kit (Roche Life Science, Indianapolis, IN, USA) according to the manufacturer's instructions, with the addition of 10 μg linear acrylamide carrier (Applied Biosystems, Thermo Fisher Scientific). The sequencing library was prepared with the ScriptSeq v2 RNA-Seq Library Preparation Kit (Epicentre,), according to the manufacturer's instructions, and purified with the Agencourt AMPure XP PCR purification system (Beckman Coulter, Atlanta, GA, USA). Because of insufficient amplification, the library was reamplified with AccuPrime *Pfx* DNA polymerase (Life Technologies, Carlsbad, CA, USA) and P5 and P7 sequence primers.

Sequencing

Paired-end sequencing (2 ×100 bp) was performed on the Illumina HiSeq 2000 platform at BGI-Europe (DK-2200 Copenhagen N, Denmark).

Sequence Data Analysis

Paired-end sequencing reads were trimmed of adaptor sequences, and overlapping read pairs were merged by using AdapterRemoval (*4*) (version 1.5.3). Reads shorter than 30 nt after trimming were excluded from further analysis. For filtering of human reads, the remaining reads were mapped to the human genome (hg38) by using the mem algorithm implemented in the Burrows-Wheeler Aligner, version 0.7.7 (BWA, http://bio-bwa.sourceforge.net/) (*5*). Reads of a pair were evaluated independently. Reads containing 25 bp or more of low complexity regions were filtered out by the DustMasker algorithm (*6*) (version 1.0.0). Filtered reads were assembled de novo with IDBA (*7*) (version 1.1.1) with default parameters. Contigs were aligned to sequences in the NCBI nucleotide database (nt) by BLASTn (megablast) (*8*) with a cutoff e-value of 10^{-3}.

Assembly of the Cutavirus Genome

BLASTn analysis initially identified 8 contigs aligning to bufavirus-1 or −2. Of these, 3 contigs were detected in the dataset from circular DNA enrichment, and 5 were detected in the

dataset from the virion-enriched sample. Subsequent analysis revealed that the contigs had high similarity to those of he recently reported cutaviruses. The near complete genome of CutaV CGG5–268 was assembled from the contigs by using Geneious 7.1.7 software (Biomatters Limited, http://www.geneious.com/). The filtered reads were mapped back to the obtained genome with BWA. From the circular enrichment and virion-enriched datasets, 7,070 and 1,332 unique reads, respectively, could be mapped back to the genome, yielding a mean depth of coverage of 191. Ambiguous bases were corrected based on the mapped reads.

Phylogenetic Analysis

Phylogenetic analysis was performed by aligning the amino acid sequences of the NS1 or VP1 protein for the cutavirus strains having full-length sequences and 1 representative for each of the 3 bufavirus genotypes. Gray fox amdovirus was used as the outgroup. The sequences were aligned by using Clustal Omega, version 1.2.2 (EMBL-EBI, http://www.ebi.ac.uk/Tools/msa/clustalo/). The phylogenetic tree was built by the maximum likelihood method with 100 bootstrap replicates. The tree was visualized with MEGA7 software (*9*).

Real-Time PCR

Real-time PCR of cutavirus was performed on total DNA extracts or sequencing libraries prepared from total DNA extracts with the LightCycler 480 Probes Master reagents (Roche), including 500 nM target specific primers and 200 nM fluorescently labeled probes (Table 1), 1–2.5 μL template (Tables 2–3), and H_2O to a final volume of 25 μL. Beta-2 microglobulin (B2M) primers and probes (Table 1) were used as a positive control. All reactions were run in duplicates. PCR cycling conditions were as follows: initial denaturation at 95°C for 10 min, followed by 45 amplification cycles of 95°C for 10 s and 60°C for 1 min.

Two rounds of PCR were run. In the first run, cutavirus primers were run with extracts from 7 of the 10 melanoma samples, including the sample in which the cutavirus contigs were identified (CGG5–268), and with a sequencing library prepared from total DNA extract from the cutavirus sample. B2M primers were run with one of the extracts as a positive control for the assay. Both the extract and library originating from the cutavirus sample tested positive, as did the B2M positive control (Figure 1). In the second run, cutavirus primers were run with libraries originating from the remaining 3 melanoma samples, for which no total DNA extract remained.

The cutavirus-positive sample was also included. B2M primers were run with the libraries as well, as a positive control. As in the first round, only the library from the cutavirus sample tested positive with the cutavirus primers, whereas all libraries tested positive with the B2M primers (Figure 2).

References

1. Hansen TA, Fridholm H, Frøslev TG, Kjartansdóttir KR, Willerslev E, Nielsen LP, et al. New type of papillomavirus and novel circular single stranded DNA virus discovered in urban *Rattus norvegicus* using circular DNA enrichment and metagenomics. PLoS One. 2015;10:e0141952. http://dx.doi.org/10.1371/journal.pone.0141952

2. Mollerup S, Friis-Nielsen J, Vinner L, Hansen TA, Richter SR, Fridholm H, et al. Propionibacterium acnes: disease-causing agent or common contaminant? Detection in diverse patient samples by next-generation sequencing. J Clin Microbiol. 2016;54:980–7. http://dx.doi.org/10.1128/JCM.02723-15

3. Jensen RH, Mollerup S, Mourier T, Hansen TA, Fridholm H, Nielsen LP, et al. Target-dependent enrichment of virions determines the reduction of high-throughput sequencing in virus discovery. PLoS One. 2015;10:e0122636. http://dx.doi.org/10.1371/journal.pone.0122636

4. Lindgreen S. AdapterRemoval: easy cleaning of next-generation sequencing reads. BMC Res Notes. 2012;5:337. http://dx.doi.org/10.1186/1756-0500-5-337

5. Li H, Durbin R. Fast and accurate long-read alignment with Burrows-Wheeler transform. Bioinformatics. 2010;26:589–95. http://dx.doi.org/10.1093/bioinformatics/btp698

6. Morgulis A, Gertz EM, Schäffer AA, Agarwala R. A fast and symmetric DUST implementation to mask low-complexity DNA sequences. J Comput Biol. 2006;13:1028–40. http://dx.doi.org/10.1089/cmb.2006.13.1028

7. Peng Y, Leung HCM, Yiu SM, Chin FYL. IDBA-UD: a de novo assembler for single-cell and metagenomic sequencing data with highly uneven depth. Bioinformatics. 2012;28:1420–8. http://dx.doi.org/10.1093/bioinformatics/bts174

8. Altschul SF, Gish W, Miller W, Myers EW, Lipman DJ. Basic local alignment search tool. J Mol Biol. 1990;215:403–10. http://dx.doi.org/10.1016/S0022-2836(05)80360-2

9. Kumar S, Stecher G, Tamura K. MEGA7: Molecular Evolutionary Genetics Analysis Version 7.0 for Bigger Datasets. Mol Biol Evol. 2016;33:1870–4. http://dx.doi.org/10.1093/molbev/msw054

Technical Appendix Table 1. Primer sequences used in real-time PCR of cutavirus.

Target	Primer	Sequence
CutaV CGG5–268	Forward primer	CAGCCATGAAATACCAACCA
	Reverse primer	CCAATTTTCTCCCCAAGTAGG
	Probe	FAM-CATAGAAAGATGGGAAACCACG-BHQ1
B2M	Forward primer	CAAATCCCACTGTCACATGCA
	Reverse primer	TGGTTGAGTTGGACCCGATAA
	Probe	HEX-TCCCATTTGCCATAGTCCTCACCTATCCCT-BHQ1

Technical Appendix Table 2. Samples tested in first-round real-time PCR of cutavirus*

Well	Well name†	Volume used, μL	Dye	Ct value
G6	Cutavirus; CGG5–268 extract	1	FAM	28.9
H6	Cutavirus; CGG5–268 extract	<1‡	FAM	27.51
A4	Cutavirus; CGG5–268 library	1	FAM	30.64
B4	Cutavirus; CGG5–268 library	1	FAM	30.43
A10	B2M; CGG5–267 extract	2.5	HEX	28.26
B10	B2M; CGG5–267 extract	2.5	HEX	28.25
A2	Cutavirus; CGG5–260 extract	2.5	FAM	No Ct
B2	Cutavirus; CGG5–260 extract	2.5	FAM	No Ct
G2	Cutavirus; CGG5–262 extract	2.5	FAM	No Ct
H2	Cutavirus; CGG5–262 extract	2.5	FAM	No Ct
G4	Cutavirus; CGG5–265 extract	2.5	FAM	No Ct
H4	Cutavirus; CGG5–265 extract	2.5	FAM	No Ct
A6	Cutavirus; CGG5–266 extract	2.5	FAM	No Ct
B6	Cutavirus; CGG5–266 extract	2.5	FAM	No Ct
D6	Cutavirus; CGG5–267 extract	2.5	FAM	No Ct
E6	Cutavirus; CGG5–267 extract	2.5	FAM	No Ct
A8	Cutavirus; CGG5–269 extract	2.5	FAM	No Ct
B8	Cutavirus; CGG5–269 extract	2.5	FAM	No Ct
D8	Cutavirus; H_2O	2.5	FAM	No Ct
E8	Cutavirus; H_2O	2.5	FAM	No Ct
D10	B2M; H_2O	2.5	HEX	No Ct
E10	B2M; H_2O	2.5	HEX	No Ct

* B2M, beta-2 microglobulin; Ct, cycle threshold; FAM, 6-carboxyfluorescein; HEX, 6-hexachlorofluoroscein.
†Well names include primers used and sample name/type.
‡Less than 1 μL extract remained for the second replicate.

Technical Appendix Table 3. Samples tested in second round real-time PCR of cutavirus*

Well	Well name†	Template dilution	Vol. used	Dye	Ct-value
A4	Cutavirus; CGG5–268 library		1	FAM	30.13
B4	Cutavirus; CGG5–268 library		1	FAM	30.54
B8	B2M; CGG5–261 library	1:10	1	HEX	37.51
A8	B2M; CGG5–261 library	1:10	1	HEX	37.72
A10	B2M; CGG5–263 library	1:10	1	HEX	33.04
B10	B2M; CGG5–263 library	1:10	1	HEX	33.48
G8	B2M; CGG5–264 library	1:10	1	HEX	31.84
H8	B2M; CGG5–264 library	1:10	1	HEX	32.35
E8	B2M; CGG5–268 library	1:10	1	HEX	32.62
D8	B2M; CGG5–268 library	1:10	1	HEX	33.05
A2	Cutavirus; CGG5–261 library		1	FAM	No Ct
B2	Cutavirus; CGG5–261 library		1	FAM	No Ct
D2	Cutavirus; CGG5–263 library		1	FAM	No Ct
E2	Cutavirus; CGG5–263 library		1	FAM	No Ct
G2	Cutavirus; CGG5–264 library		1	FAM	No Ct
H2	Cutavirus; CGG5–264 library		1	FAM	No Ct
D4	Cutavirus; H_2O		2.5	FAM	No Ct
E4	Cutavirus; H_2O		2.5	FAM	No Ct
D10	B2M; H_2O		2.5	HEX	No Ct
E10	B2M; H_2O		2.5	HEX	No Ct

*B2M, beta-2 microglobulin; Ct, cycle threshold; FAM, 6-carboxyfluorescein; HEX, 6-hexachlorofluoroscein; vol., volume.
†Well names include primers used and sample name/type.
.

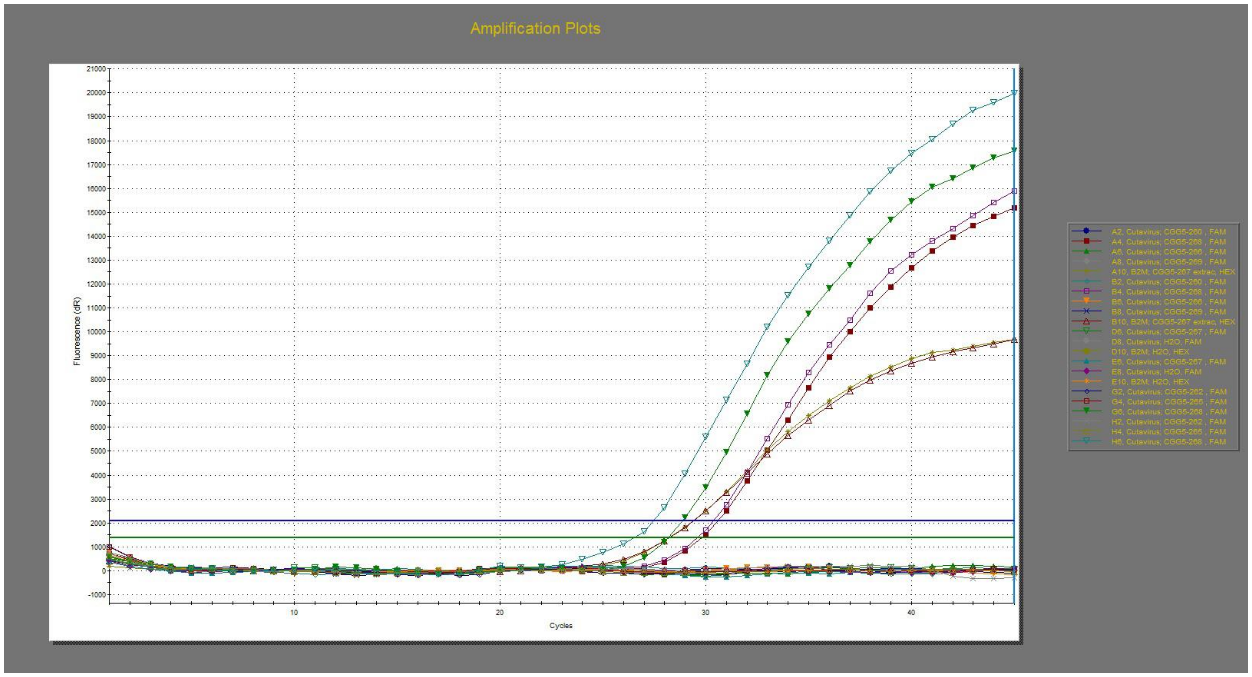

Technical Appendix Figure 1. Amplification curves for the first round of real-time PCR of cutavirus.

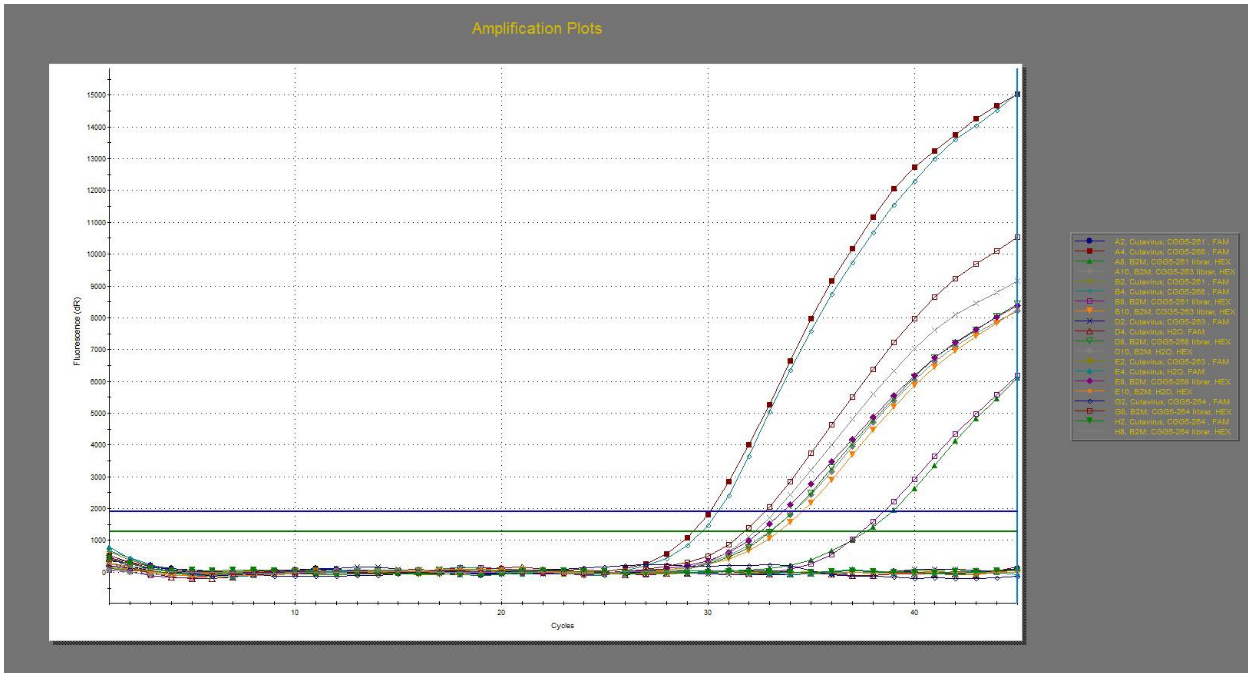

Technical Appendix Figure 2. Amplification curves for the first round of real-time PCR of cutavirus. All curves below the “B2M” represent libraries run with B2M primers.
